# Twice perforated stump appendicitis: a case report

**DOI:** 10.11604/pamj.2022.43.43.37149

**Published:** 2022-09-27

**Authors:** Pueya Rashid Nashidengo, Francis William Quayson, John Tabiri Abebrese, Rejoice Tjipetekera, Sharifa Sushmita Seibes

**Affiliations:** 1 Department of Surgery, Windhoek Central Hospital, Windhoek, Namibia

**Keywords:** Appendicitis, stump appendicitis, exploratory laparotomy, case report

## Abstract

Stump appendicitis is an acute inflammation of the residual appendix and one of the rare complications after appendectomy. Stump appendicitis is an under-reported and poorly defined condition related to obstruction and inflammation of the residual appendix after an appendectomy, usually by a fecolith. It remains a clinical challenge because of delayed diagnosis and subsequent treatment with increased morbidity or mortality. Herein, we describe the case of a 42-year-old male who presented with periumbilical pain with progression to generalized abdominal pain and signs of peritonitis 14 months post appendectomy. An exploratory laparotomy revealed an inflamed, non-gangrenous perforated appendices stump. We discuss the challenges in the diagnosis and management thereof.

## Introduction

Appendectomy is one of the most commonly performed surgical procedures involving resection of the vermiform appendix at its base, located at the confluence of the taenia coli, posteromedial to the cecal wall [1]. In rare cases, the organ is not entirely resected, resulting in a residual appendix, potentially developing into stump appendicitis (SA) [2]. Stump appendicitis is one of the rare delayed complications post appendectomies, with a reported incidence of 1 in 50,000 cases. Prompt recognition is vital for early treatment, thus avoiding complications like perforation of the appendix with resultant peritonitis, intra-abdominal abscess formation, sepsis, and death. [3]. Patients with stump appendicitis generally present with acute appendicitis with associated localized or generalized peritonitis. They may also present with an appendices mass, appendices abscess, or ileum. In patients with a surgical history of a previous appendectomy, stump appendicitis may not be considered a differential diagnosis with consequent delay in diagnosis. This clinical challenge places patients at an increased mortality and morbidity risk [4]. We present the case of a 42-year-old male who underwent open appendectomy and presented 14 months post-index surgery with a history of abdominal pain with distension, anorexia, vomiting, and constipation. On examination, he had signs of peritonitis. An assessment of an acute abdomen was made, with a differential diagnosis of complicated adhesive small bowel obstruction. An emergency exploratory laparotomy was performed with findings of an inflamed and perforated appendices stump and pus contamination of the left and right lower quadrants.

## Patient and observation

**Patient information:** a 42-year-old male presented to the emergency department with a chief complaint of a three-day history of severe periumbilical pain that was initially intermittent and localized, then gradually progressed to constant and generalized. In addition, the patient complained of constipation, bilious vomiting, and gradual abdominal distension that had started after the onset of the pain. However, there was no associated fever. His surgical history was significant for a previous emergency open appendectomy via a midline incision performed 14 months prior to his current presentation with intraoperative findings of a perforated appendix with four-quadrant pus contamination. In addition, his medical history was significant for hypertension diagnosed a year prior and fairly controlled on antihypertensive. His vital readings revealed a heart rate of 117 beats per minute and blood pressure of 140/104mmHg.

**Clinical findings:** physical examination of the abdomen revealed generalized abdominal distension and a healed lower midline scar. Abdominal palpation revealed generalized tenderness, rebound tenderness, guarding, and rigidity. The rest of the systemic examination was unremarkable.

**Diagnostic assessment:** routine laboratory investigations revealed a total white cell count (WCC) of 5.97 x 109/L (normal range of 3.80 - 8.76 x 109), neutrophils of 75.5%, c-reactive protein (CRP) of 442.1 mg/l (normal range of 0 - 10mg/l), urea of 13.8 (normal range 2.1-7.1 mmol/l) and creatinine of 124umol/l (normal range of 26.5 - 88.4umol/l). A plain abdominal X-ray demonstrated centrally located dilated bowel loops with valvulae coniventes and centrally located multiple air-fluid levels.

**Figure 1:**
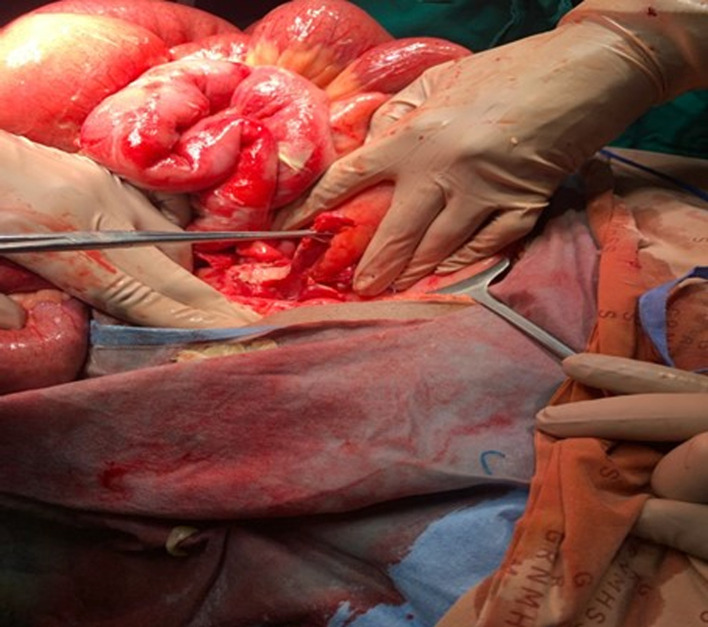
intra-operative picture of a perforated non-gangrenous appendiceal stump

**Therapeutic intervention:** the patient was kept nil per os. A nasogastric tube and urinary catheter were inserted. Intravenous fluids (crystalloids), parenteral antibiotics, and analgesia were initiated. Consent was obtained for an emergency exploratory laparotomy. The abdomen was entered via a lower midline incision with findings of dilated loops of bowel and pus in the right and left lower quadrants of the abdomen. Further exploration revealed an inflamed, non - gangrenous perforated appendices stump about 5 cm long (Figure 1). The appendices stump was ligated at the base, and a thorough peritoneal washout was performed. Broad-spectrum antibiotics were continued postoperatively.

**Timeline:** the patient underwent an appendectomy via lower midline incision 14 months before the presentation. He presented with a three-day history of peri-umbilical pain that progressed to generalized pain. He also presented with constipation, bilious vomiting, and gradually progressive abdominal distension. On examination, he had generalized abdominal tenderness, rebound tenderness and guarding. The investigations included a complete blood count, serum urea and electrolytes, c-reactive protein, and plain abdominal X-rays. The patient underwent an emergency laparotomy with findings of stump appendicitis. The appendices stump was perforated, the small and large bowel loops were dilated due to the ileus, and there was pus in the abdomen. The postoperative period was unremarkable, with the patient mainly complaining of pain at the incision site. The patient was discharged six days post admission.

**Pathology report:** specimen from recent surgery: microscopically, the appendix was thickened with a size of 5 cm by 1 cm. Microscopically the appendiceal sections showed congestion with transmural mixed inflammatory cell infiltrates. There were no parasites or neoplasm. These features were consistent with acute appendicitis with perforation and peritonitis. Specimen from index surgery: microscopically, the appendix was 3 cm by 1 cm with partially ulcerated mucosa and transmural mixed inflammation. The serosa was congested, and no parasites or neoplasms were identified. The features were consistent with appendicitis with peritonitis.

**Follow-up and outcome of interventions:** the patient was discharged six days post-surgery, followed up for wound care on an outpatient basis, and fully recovered.

**Patient perspective:** according to the patient, he is feeling better and has returned to his pre-surgical level of functionality.

**Informed consent:** we obtained consent from the patient for the publication of this article.

## Discussion

Stump appendicitis (SA) is the interval of repeated inflammation of remaining residual appendices tissue after an appendectomy. Incompletely removing an appendix leaves a stump behind, allowing for recurrent appendicitis [5]. Stump appendicitis was first described by TF Rose in 1945, followed by Baumgardner in 1949 [6]. With a reported frequency of 1 in 50 000, SA is a rare, delayed complication of appendicitis. It presents a diagnostic dilemma in patients with a previous appendectomy [7]. The risk of complications such as perforation rises due to the delay in diagnosis. In a retrospective study of 3130 patients who had appendectomies, five had stump appendicitis, four of which had a ruptured appendix [2]. Perforation is common in individuals with stump appendicitis, with up to 70% of cases perforating, as demonstrated in this case report [8]. According to Kanona *et al*. literature study, the age range for 51 documented cases of stump appendicitis was 8-72 years old, with 67 percent of the patients being male. The symptoms of stump appendicitis are identical to those of acute appendicitis [2]. Right lower quadrant discomfort (59 percent), nonspecific abdominal pain (16 percent), and central abdominal pain spreading to the lower quadrant were the most prevalent complaints (14 percent) [9]. Stump appendicitis can cause intestinal obstruction in 1-5 percent of cases. The appendicular infection and inflammation can irritate neighboring bowel, culminating in paralytic ileus [10]. The symptoms of small bowel obstruction were evident in our case. Stump appendicitis has been recorded to occur between three weeks to twenty-three years after an appendectomy. Our case was 14 months after an open appendectomy.

The most common surgical error associated with stump appendicitis is the failure to detect the appendices base, resulting in a long appendices stump [8]. Stump appendicitis is linked to the surgeon's clinical experience, significant appendices' inflammation, and the appendix's anatomical position. During appendectomy, the intense inflammation impairs the detection of the appendix's sub-serosal position, making it difficult to isolate. Dikicier *et al*. reported three out of five patients with a subserosal appendix, while the other two had residual appendices tissue with a 5.9 cm remaining appendices stump length [2]. This is consistent with Kanona *et al*. findings, which indicated an appendices stump range of 0.5 cm to 6.5 cm [8]. The patient had a residual appendix of 5 cm. Reports have shown that patients who had a laparoscopic appendectomy have a higher risk of developing SA. However, Liang *et al*. found that only 34% of the 36 cases they studied had an initial laparoscopic appendectomy [10]. This is consistent with the findings of Geraci *et al*., who discovered that the first appendectomy was open in 65 percent of SA cases [4]. Preoperative stump appendicitis is still diagnosed clinically because patients often present with signs and symptoms similar to acute appendicitis. Plain radiography, ultrasound, and computed tomography may all be used to help diagnose it, particularly in cases where there is an abscess or a perforation with intraperitoneal fluid collecting in the right lower abdomen or pelvis [8].

## Conclusion

Stump appendicitis presents a challenge for surgeons because it is rare, and delaying diagnosis results in considerable morbidity. Nevertheless, it should remain a differential diagnosis for patients who have had an appendectomy and are experiencing right lower quadrant pain.
